# Supplementary material for: The short isoform of the host antiviral protein ZAP acts as an inhibitor of SARS-CoV-2 programmed ribosomal frameshifting
Source: Nat Commun. 2021 Dec 10;12:7193. doi: 10.1038/s41467-021-27431-0 (PMC8664833; doi:10.1038/s41467-021-27431-0)
Supplement: Supplementary file 1 — Supplementary Information [file 41467_2021_27431_MOESM1_ESM.pdf]

Supplementary Information for

The short isoform of the host antiviral protein ZAP acts as an inhibitor of SARS-CoV-2 programmed ribosomal frameshifting

Matthias M. Zimmer[1,†], Anuja N. Kibe[1,†], Ulfert Rand[2], Lukas Pekarek[1], Liqing Ye[1], Stefan Buck[1], Redmond Smyth[1,3], Luka Cicin-Sain[2], Neva Caliskan[1,3,*]

[1] Helmholtz Institute for RNA-based Infection Research (HIRI), Helmholtz Zentrum für Infektionsforschung (Helmholtz Centre for Infection Research), Josef-Schneider-Strasse 2, 97080, Würzburg, Germany

[2] Helmholtz Zentrum für Infektionsforschung, Inhoffenstrasse 7, 38124, Braunschweig, Germany

[3] Medical Faculty, Julius-Maximilians University Würzburg, 97074, Würzburg, Germany

[†] These authors contributed equally to this work.

[*]Corresponding author. E-Mail: neva.caliskan@helmholtz-hiri.de

This PDF file includes:

Supplementary Figures 1-6

Supplementary Tables 1-3

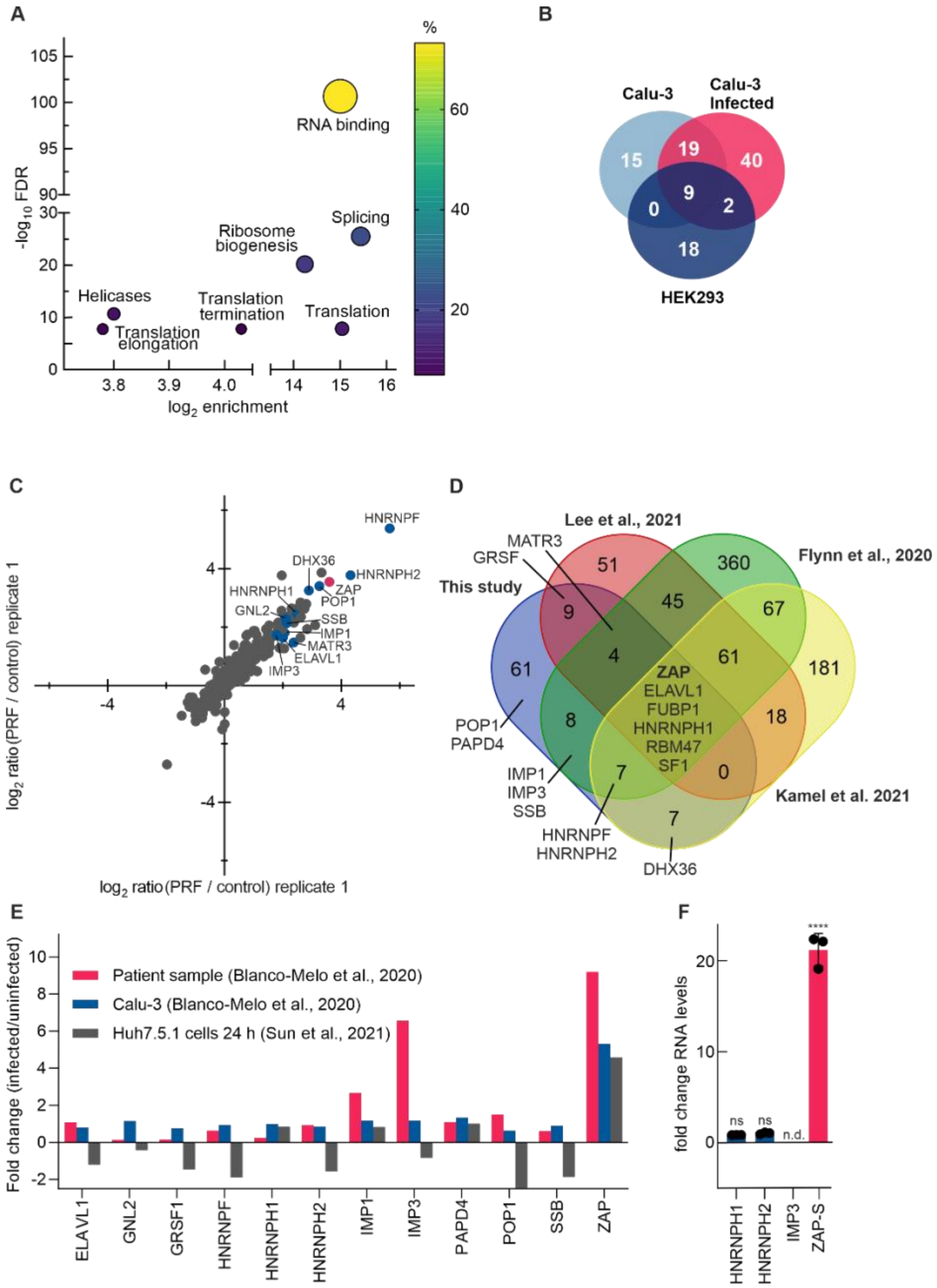

Supplementary Fig. 1. Capture and analysis of frameshift RNA interactors, related to Fig. 1. (A) Gene ontology (GO) term analysis of SARS-CoV-2 frameshift RNA interactions. FDR – false discovery rate. **(B)** Venn Diagram comparing the hits of the *in vitro* RNA antisense purification in HEK293, uninfected Calu-3 as well as SARS-CoV-2 infected Calu-3 cells. **(C)** Scatter plot of log2-transformed ratio of RNA-antisense purification in HEK293 cells. **(D)** Venn Diagram comparing the hits of the vitro RNA antisense purification of the SARS-CoV-2 frameshift site from this study with the hits of genome-wide interactome captures from the literature [1,2,3]. **(E)** Fold change of SARS-CoV-2 PRF element interactors in published RNAseq datasets. **(F)** Expression profiles of selected genes in Calu-3 infected cells with SARS-CoV-2 at 72 hours. RNA levels were quantified by qRT-PCR and normalized to the respective RNA abundance in uninfected cells (shown as $2^{-\Delta\Delta CT}$). Data points represent the mean ± s.d. (n = 3 independent experiments). P values were calculated using an ordinary unpaired one-sided ANOVA comparing ΔC_t values of the respective RNA in uninfected and infected cells. Exact p values: ZAP-S – <0.0001. n.d. - not detected. See also **Supplementary Table 2**.

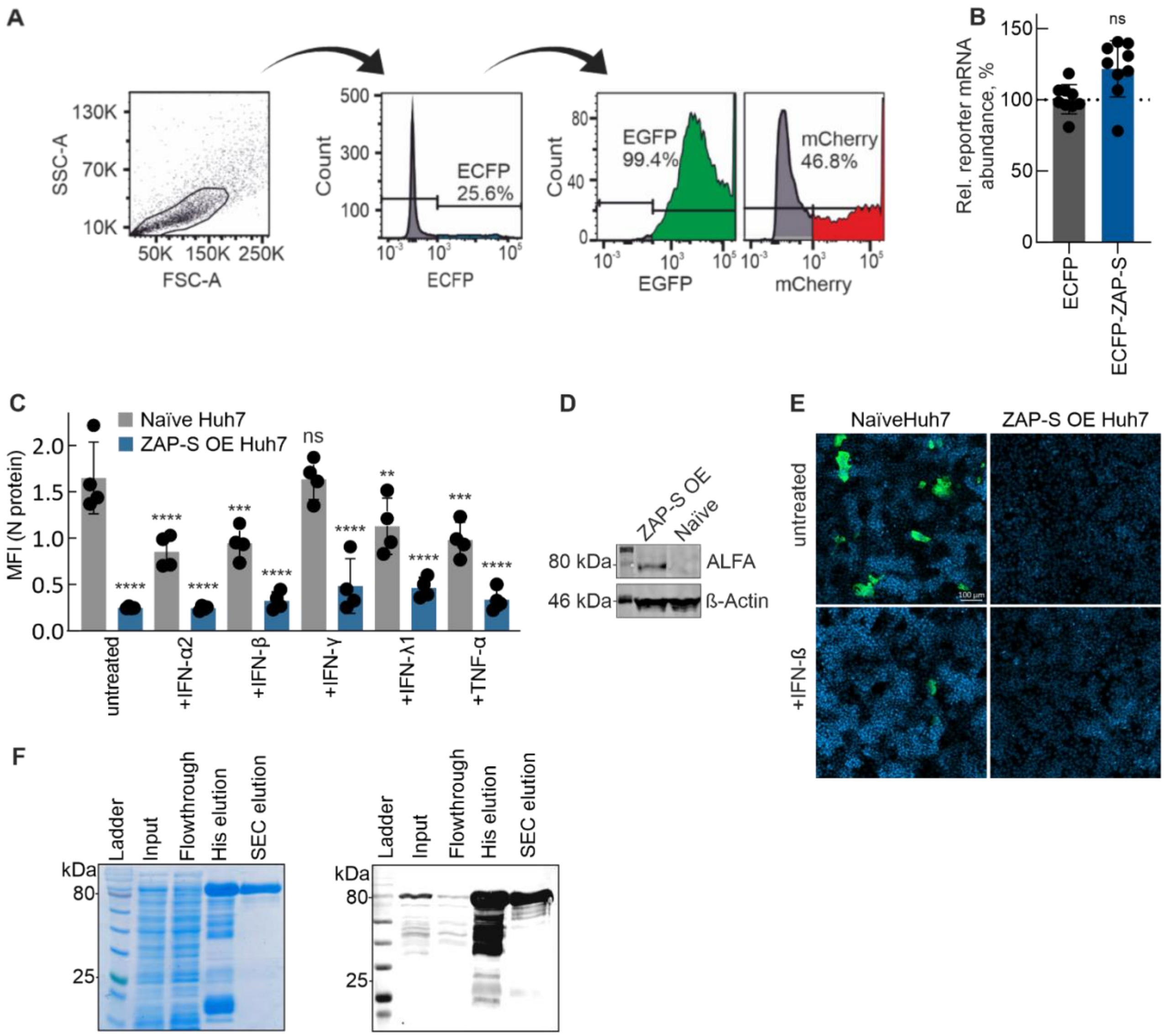

Supplementary Fig. 2. Effect of ZAP-S on FE of various PRF sites, related to Fig. 2 and 3. (A) Gating strategy for flow cytometry determining FE in HEK293 cells. Cell populations were determined based on SSC and FSC. ECFP-positive cells were analyzed for the mean intensities of EGFP and mCherry. **(B)** Abundance of reporter mRNA upon overexpression of ZAP-S determined by qRT-PCR. Datapoints represent the mean ± s.d. (n = 3 independent experiments with three technical replicates each). P values were calculated using an ordinary unpaired one-sided ANOVA comparing raw Ct values to the ECFP control. Exact P values: **** P < 0.0001, Naïve Huh7 IFN-β – 0.0003, Naïve Huh7 IFN-λ1 – 0.0086, Naïve Huh7 TNF-α – 0.0005. **(C)** Quantification of immunostaining of N-protein in infected naïve Huh7 or ZAP-S overexpressing Huh7 cells (ZAP-S OE) at 24 hours post-infection. Treatment with IFN-γ (500 U/ml), IFN-β (500 U/ml), or IFN-λ1 (5 ng/ml) was done one hour before infection. Boxes show mean values ± s.d. (n = 4 technical replicates). **(D)** Western blot of naïve as well as ALFA-tagged ZAP-S-overexpressing Huh7 cells. ALFA-ZAP-S was detected using anti-ALFA antibody, ß-actin serves as a loading control. n = 3 independent experiments. **(E)** Confocal micrograph of immunostaining of N-protein in infected naïve Huh7 or ZAP-S overexpressing Huh7 cells (ZAP-S OE) at 24 hours post-infection. **(F)** Coomassie-stained SDS-PAGE and western blot of heterologous expression of ZAP-S in *E. coli* as well as the purification steps. ZAP-S was detected using an anti-ZC3HAV1 (ZAP) antibody. n = 1 experiment.

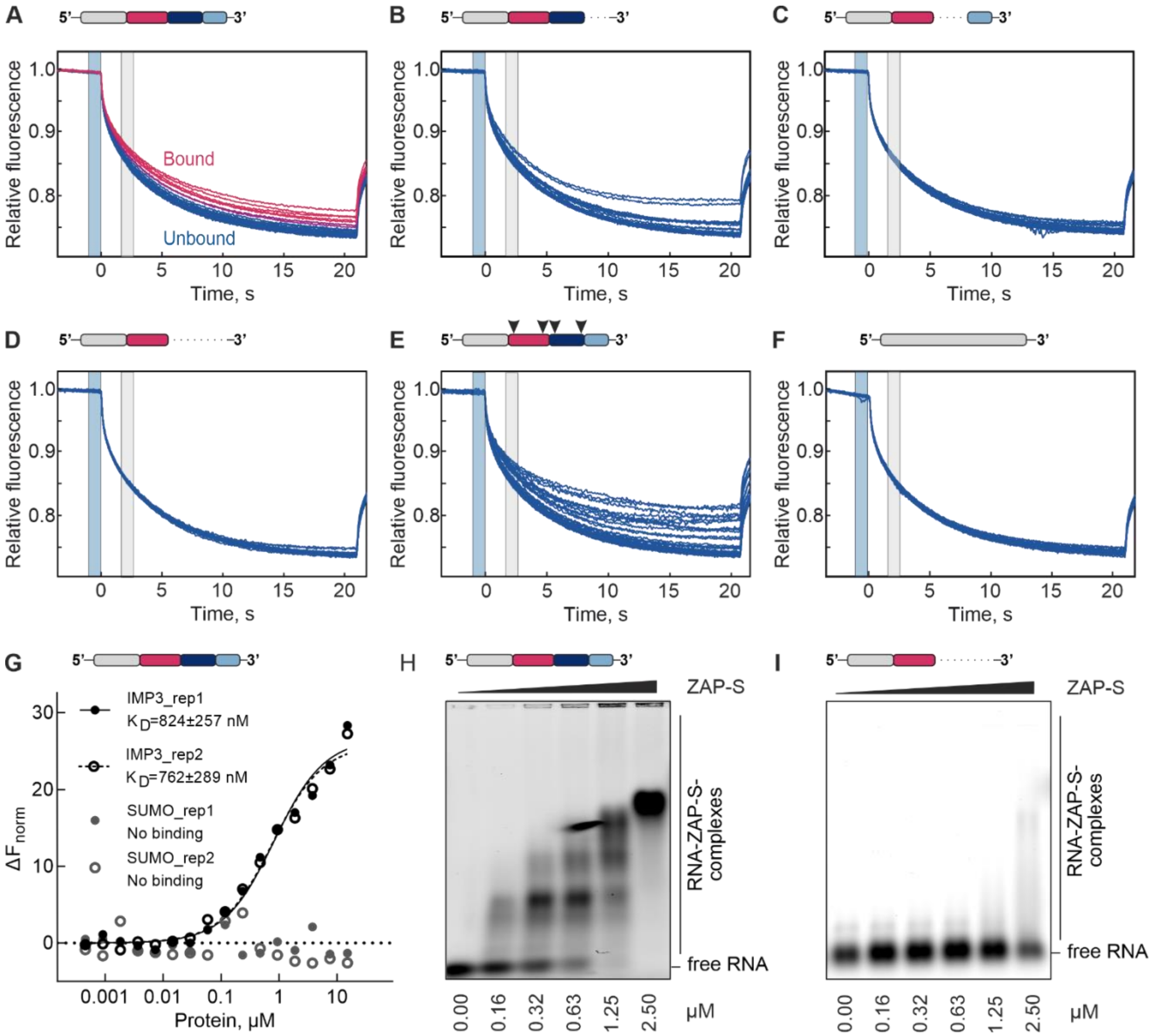

Supplementary Fig. 3. Thermophoresis raw data and interaction of IMP3 and SUMO with SARS-CoV-2 FS RNA, related to Fig. 4. Thermophoretic time-traces of microscale thermophoresis (MST) measurements of binding between ZAP-S and **(A)** Full PRF, **(B)** ΔSL2 mutant, **(C)** ΔSL3 mutant, **(D)** ΔSL2+3 mutant, **(E)** compensatory mutant and **(F)** scrambled mutant. Blue and grey boxes in the time-course traces represent the temperature jump and MST-on time (2.5 s), respectively. In all cases, there is no adsorption of the labeled protein to the capillaries. See Fig. 4 for resulting binding curves. **(G)** Microscale thermophoresis to monitor binding of IMP3 and SUMO to SARS-CoV-2 FS PK. Unlabeled protein (0.4 nM to 15 µM) was titrated against 3' pCp-Cy5 labeled RNA (5 nM) and thermophoresis was recorded at 25°C with 5% LED intensity and medium MST power. Change in fluorescence (ΔF_{norm}) was measured at MST on-time of 2.5 s. Data were analyzed using standard functions in the MO. Affinity Analysis software and K_D was determined using Graphpad Prism 9.2.0. Data represent mean +/- s.d. of each independent measurement (n=2 independent experiments). Electrophoretic mobility Assay (EMSA) of **(H)** SARS-CoV-2 pseudoknot and **(I)** ΔSL2+3 mutant. 100 nM RNA labelled with Cy5 at the 3'end was incubated with serial dilutions of ZAP-S in Buffer A supplemented with 5% glycerol. Reactions were separated by 0.5% agarose electrophoresis in 1x TBE prior to visualization. n = 2 independent experiments.

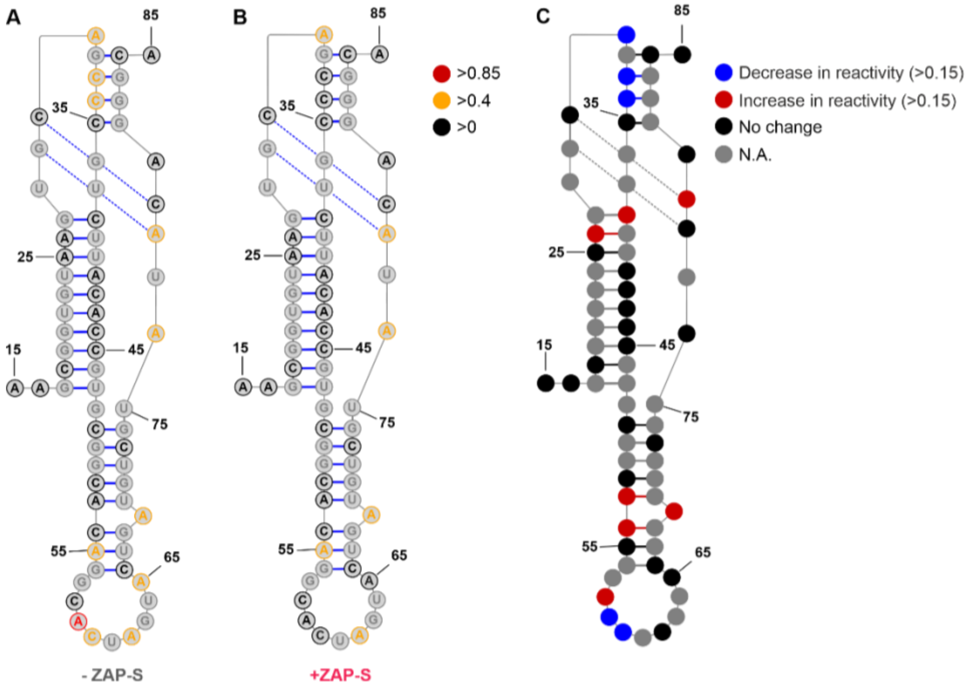

Supplementary Fig. 4. **DMS MaPseq on SARS-CoV-2 RNA**. DMS reactivities of SARS-CoV-2 RNA folding **(A)** without ZAP-S or **(B)** in the presence of ZAP-S (5 μM) **(B)**. **(C)** Changes in DMS reactivity upon ZAP-S binding. Blue nucleotides represent decrease in reactivity, and red nucleotides represent increase in reactivity.

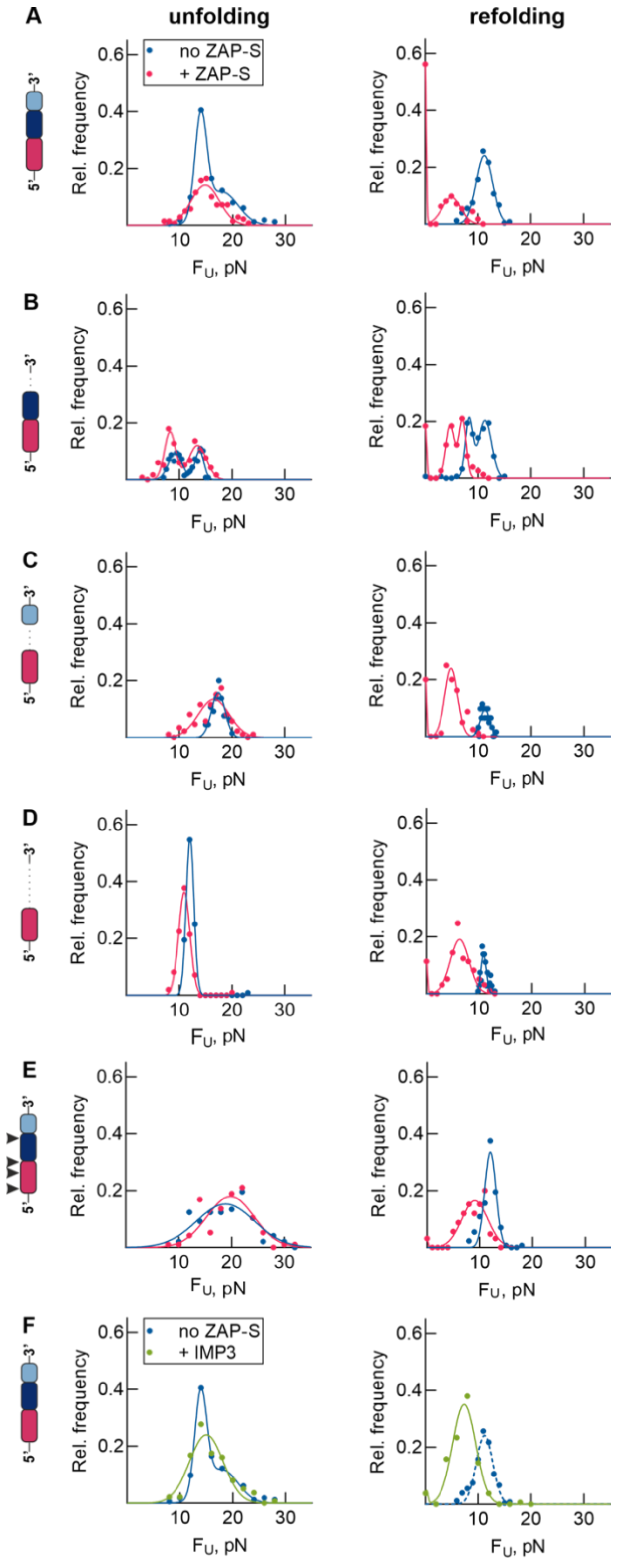

Supplementary Fig. 5. Optical tweezers data related to Fig. 5. (A-F) Distributions of unfolding and refolding force (F_U), respectively, in absence (blue) and presence (pink) of ZAP-S protein for different RNA samples measured in OT. (A) PK, (B) ΔSL2 mutant, (C) ΔSL3 mutant, (D) ΔSL2+3 mutant, (E) compensatory mutant, (F) PK in absence (blue) and presence (green) of IMP3.

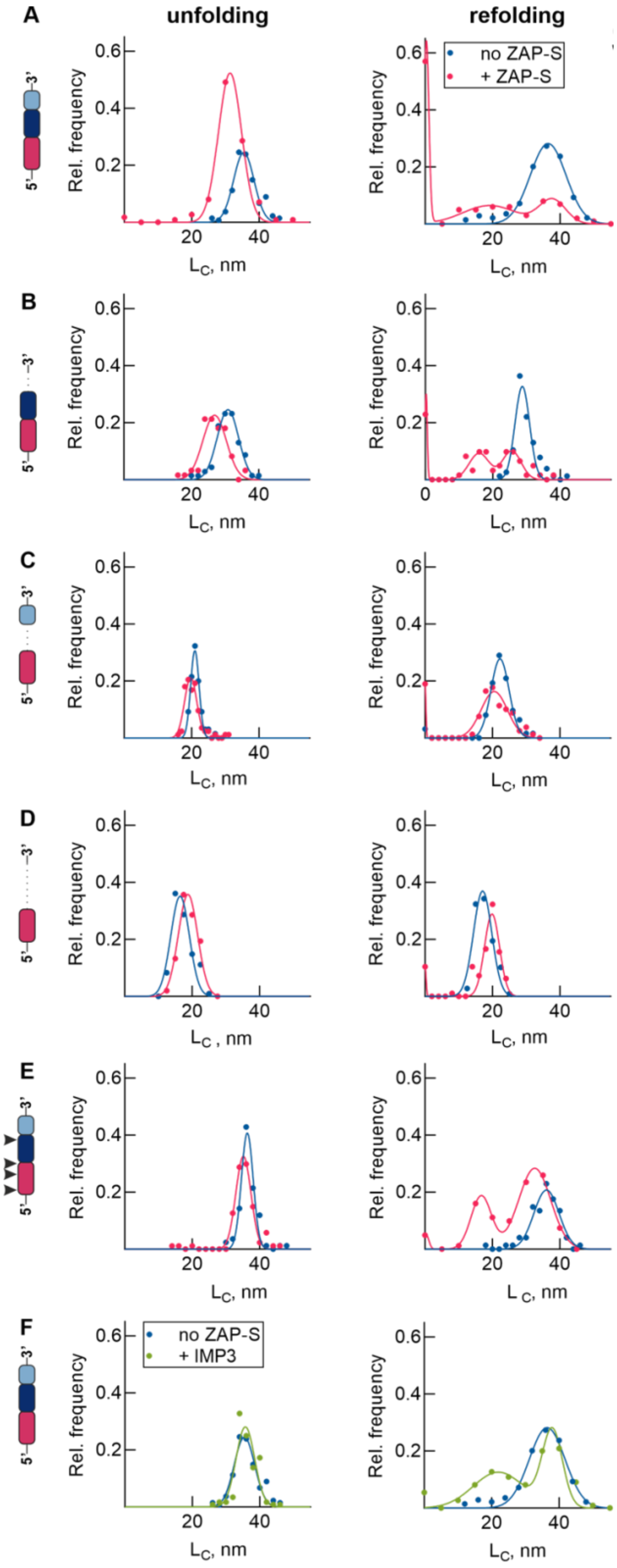

Supplementary Fig. 6. Optical tweezers data related to Fig. 5. (A-F) Distributions of unfolding and refolding contour length change (L_C), respectively, in absence (blue) and presence (pink) of ZAP-S protein for different RNA samples measured in OT. (A) PK, (B) ΔSL2 mutant, (C) ΔSL3 mutant, (D) ΔSL2+3 mutant, (E) compensatory mutant, (F) PK in absence (blue) and presence (green) of IMP3.

Supplementary Table 1: Values of fitted and calculated parameters for individual samples. Errors on the measured values represent standard error on the mean.

	Direction	Peak #	Work, kBT	Contour length, nm	Force, pN	Number of FD curves / molecules
**PK**	unfolding	1	74.1±16.6	35.4±3.0	14.0±1.1	273/24
		2			18.0±3.0	
	refolding	1	50.5±15.0	36.4±5.3	11.2±1.6	
**PK+ZAP**	unfolding	1	63.3±22.8	31.4±3.4	14.8±2.7	219/11
		2				
	refolding	1	0.0±0.9	0.4±0.7	0	
		2		19.2±8.0	4.8±1.8	
		3		37.7±3.9	9.3±0.5	
**PK+IMP3**	unfolding	1	79.2±24.2	35.7±2.8	15.0±3.2	226/20
	refolding	1	19.8±8.0	22.0±7.5	7.4±2.2	
		2		38.0±3.0		
**Δ SL2**	unfolding	1	48.9±8.0	30.8±3.1	9.3±1.3	146/8
		2			13.8±0.8	
	refolding	1	39.0±7.9	28.7±2.1	8.3±0.7	
		2			11.3±1.2	
**Δ SL2+ZAP**	unfolding	1	39.1±4.8	26.9±3.5	8.3±1.1	122/8
		2			13.5±1.7	
	refolding	1	1.4±4.0	0.2±0.3	4.8±0.8	
		2	12.9±2.6	15.9±3.1	7.1±0.7	
		3	23.1±2.7	25.6±2.8		
**Δ SL3**	unfolding	1	50.4±7.0	21.0±1.2	17.4±1.3	127/12
	refolding	1	30.3±4.4	22.2±2.7	11.3±0.9	
**Δ SL3+ZAP**	unfolding	1	39.8±13.9	19.7±1.8	16.6±2.9	163/11
	refolding	1	0.8±11.3	20.5±3.9	5.1±1.2	
**Δ SL2+3**	unfolding	1	24.7±6.0	16.4±2.8	12.1±0.7	216/8
	refolding	1	21.8±6.8	17.1±2.7	10.9±0.5	
**Δ SL2+3+ZAP**	unfolding	1	25.1±7.6	18.8±2.8	11.0±1.1	196/11
	refolding	1	9.3±7.2	19.9±2.2	6.3±1.8	
**Comp. mut**	unfolding	1	114.2±33.9	36.3±1.7	18.9±5.5	158/12
	refolding	1	56.9±12.6	36.0±3.7	12.0±1.1	
**Comp. mut+ZAP**	unfolding	1	109.3±30.5	35.2±2.2	19.8±4.5	169/16
	refolding	1	29.5±13.6	18.2±5.3	9.2±2.5	
		2		32.8±3.0		

Supplementary Table 2: DNA sequences of SARS-CoV-2 PRF element and its mutants (graphically represented in Fig. 4B) employed in this study. Nucleotides in bold represent the slippery sequence and nucleotides in small letters represent mutations in the compensatory mutant.

Name	Sequence
Full PRF	TCGTT**TTTAAAC**GGGTTTGCGGTGTAAGTGCAGCCCGTCTTACACCGTGCGGCACAGGC ACTAGTACTGATGTCGTATACAGGGCTTTTGACATCTACAATGATAAAGTAGCTG
Δ SL2 mutant	TCGTT**TTTAAAC**GGGTTTGCGGTGTAAGTGCAGCCCGTCTTACACCGTGCGGCACAGGC ACTAGTACTGATGTCGTTCGTTTTTAAACGGGTTTGCGGTGTAAGTGCAGCCCGTCTTAC ACCGTGCGGCACAGGCACTAGTACTGATGTCGT
Δ SL3 mutant	TCGTT**TTTAAAC**GGGTTTGCGGTGTAAGTGCAGCCCGTCTTACACCGTATACAGGGCT
Δ SL2+3 mutant	TCGTT**TTTAAAC**GGGTTTGCGGTGTAAGTGCAGCCCGTCTTACACCGT
Comp. mut	TCGTT**TTTAAAC**GGGTTTGgGGTGTAAGTGCAGCCCGTCTTACACCcTGgGGCACAGGCA CTAGTACTGATGTCcTATACAGGGCT
Scrambled	GAAGGTAGTACCAGGTTGGAGATATACAGTGTCTTGACCCCAGGTTACTGGATTTCCCT TGGGGTCTCCCAAAACTCCCTTGAAACCTGTTGTCACTGGTGGATTAGTAAGAGGTTT

Supplementary Table 3: *In vitro* translation mean PRF values of SARS-CoV-2 in presence of ZAP-S, IMP3 or SUMO protein. Errors on the measured values represent standard error on the mean. n = 3 independent experiments.

	**Mean PRF, %**		
**[Protein], µM**	**ZAP-S**	**IMP3**	**SUMO**
**0**	49.89±5.63	45.25±9.33	46.35±16.93
**0.5**	44.10±6.07	-	-
**0.75**	40.67±7.51	40.67±6.37	48.93±14.2
**1**	38.18±6.81	49.20±1.87	45.43±9.32
**1.5**	34.07±1.41	41.11±6.90	41.63±15.53
**2**	29.29±5.14	46.31±6.13	44.68±16.03
**3**	26.18±1.03	44.85±6.62	47.30±19.81

SUPPLEMENTARY REFERENCES

1. Castello, A. *et al.* Insights into RNA biology from an atlas of mammalian mRNA-binding proteins. *Cell* **149**, 1393–1406 (2012).
2. Lee, S. *et al.* The SARS-CoV-2 RNA interactome. *Molecular Cell* **81**, 2838-2850.e6 (2021).
3. Flynn, R. A. *et al.* Discovery and functional interrogation of SARS-CoV-2 RNA-host protein interactions. *Cell* **184**, 2394-2411.e16 (2021).
