# Supplementary material for: The short isoform of the host antiviral protein ZAP acts as an inhibitor of SARS-CoV-2 programmed ribosomal frameshifting
Source: Nat Commun. 2021 Dec 10;12:7193. doi: 10.1038/s41467-021-27431-0 (PMC8664833; doi:10.1038/s41467-021-27431-0)
Supplement: Supplementary file 3 — Reporting summary [file 41467_2021_27431_MOESM3_ESM.pdf]

nature research

Corresponding author(s): Dr. Neva Caliskan

Last updated by author(s): Nov 16, 2021

Reporting Summary

Nature Research wishes to improve the reproducibility of the work that we publish. This form provides structure for consistency and transparency in reporting. For further information on Nature Research policies, see our Editorial Policies and the Editorial Policy Checklist.

Statistics

For all statistical analyses, confirm that the following items are present in the figure legend, table legend, main text, or Methods section.

n/a	Confirmed	
☐	☒	The exact sample size (*n*) for each experimental group/condition, given as a discrete number and unit of measurement
☐	☒	A statement on whether measurements were taken from distinct samples or whether the same sample was measured repeatedly
☐	☒	The statistical test(s) used AND whether they are one- or two-sided<br>*Only common tests should be described solely by name; describe more complex techniques in the Methods section.*
☐	☒	A description of all covariates tested
☐	☒	A description of any assumptions or corrections, such as tests of normality and adjustment for multiple comparisons
☐	☒	A full description of the statistical parameters including central tendency (e.g. means) or other basic estimates (e.g. regression coefficient) AND variation (e.g. standard deviation) or associated estimates of uncertainty (e.g. confidence intervals)
☐	☒	For null hypothesis testing, the test statistic (e.g. *F*, *t*, *r*) with confidence intervals, effect sizes, degrees of freedom and *P* value noted<br>*Give P values as exact values whenever suitable.*
☒	☐	For Bayesian analysis, information on the choice of priors and Markov chain Monte Carlo settings
☒	☐	For hierarchical and complex designs, identification of the appropriate level for tests and full reporting of outcomes
☒	☐	Estimates of effect sizes (e.g. Cohen's *d*, Pearson's *r*), indicating how they were calculated

Our web collection on statistics for biologists contains articles on many of the points above.

Software and code

Policy information about availability of computer code

Data collection	Optical tweezers data were collected using commercial device C-Trap (Lumicks) provided with the Bluelake software (Lumicks)<br>MST data collected using Nanotemper Monolith<br>Flow cytometry - FACSAria III (BD Biosciences) or a NovoCyte Quanteon (ACEA) instrument.<br>In vitro translation- ImageJ
Data analysis	Optical tweezers (OT) data were analyzed using a custom-written python script (available at https://github.com/lpekarek/POTATO (manuscript in preparation));<br>Data were further processed using Microsoft Excel;<br>Graphpad prism 9 software (https://www.graphpad.com/scientific-software/prism/);<br>MST: MO. Affinity Analysis software Nanotemper technologies - Visualized in Graphpad<br>Flow cytometry: Mean data exported to Excel and FE calculated with Excel - Visualized in Graphpad<br>In vitro translation: Intensities from ImageJ were exported to Excel-FE calculated in Excel. Visualized in Graphpad

For manuscripts utilizing custom algorithms or software that are central to the research but not yet described in published literature, software must be made available to editors and reviewers. We strongly encourage code deposition in a community repository (e.g. GitHub). See the Nature Research guidelines for submitting code & software for further information.

Data

Policy information about availability of data

All manuscripts must include a data availability statement. This statement should provide the following information, where applicable:

- Accession codes, unique identifiers, or web links for publicly available datasets
- A list of figures that have associated raw data
- A description of any restrictions on data availability

All raw data associated with Fig.1, Supplementary Fig. 1A-C, E and F, Fig. 2, Supplementary Fig 2B, D and F, Fig. 3B, Fig 4, Supplementary Fig. 3G-I, are provided in the Source Data File. Additional data associated with force spectroscopy and DMS-Seq are deposited in Mendeley Data: DOI: 10.17632/c7rbxb86k2.1.
The mass spectrometry proteomics data have been deposited to the ProteomeXchange Consortium via the PRIDE partner repository with the dataset identifier PXD029656.

Field-specific reporting

Please select the one below that is the best fit for your research. If you are not sure, read the appropriate sections before making your selection.

☒ Life sciences ☐ Behavioural & social sciences ☐ Ecological, evolutionary & environmental sciences

For a reference copy of the document with all sections, see nature.com/documents/nr-reporting-summary-flat.pdf

Life sciences study design

All studies must disclose on these points even when the disclosure is negative.

Sample size	n=3 for flow cytometry, MST, in vitro translation studies, which is the standard sample size for statistically significant biological tests. For the optical tweezer studies >40 which is the standard size for optical tweezer studies
Data exclusions	For the optical tweezers- curves were excluded based on the fitted total contour length change (above threshold corresponding to maximum single-molecule change). For the MST - outliers were excluded
Replication	All attempts at replication were successful
Randomization	none
Blinding	None

Reporting for specific materials, systems and methods

We require information from authors about some types of materials, experimental systems and methods used in many studies. Here, indicate whether each material, system or method listed is relevant to your study. If you are not sure if a list item applies to your research, read the appropriate section before selecting a response.

Materials & experimental systems

n/a	Involved in the study	
☐	☒	Antibodies
☐	☒	Eukaryotic cell lines
☒	☐	Palaeontology and archaeology
☒	☐	Animals and other organisms
☒	☐	Human research participants
☒	☐	Clinical data
☒	☐	Dual use research of concern

Methods

n/a	Involved in the study	
☒	☐	ChIP-seq
☐	☒	Flow cytometry
☒	☐	MRI-based neuroimaging

Antibodies

Antibodies used	DDDDK tag antibody Abcam ab49763<br>ZC3HAV1 polyclonal antibody Proteintech 16820-1-AP<br>Anti-beta actin antibody Abcam ab6276<br>RPS6 Polyclonal Antibody Proteintech 14823-1-AP<br>RPL4 Monoclonal Antibody Proteintech 67028-1-Ig<br>normal rabbit IgG Cell Signaling #2729<br>RyDEN Polyclonal antibody Proteintech 27865-1-AP

	IRDye® 800CW Goat anti-Rabbit IgG Odyssey 926-32211<br>IRDye® 680RD Donkey anti-Mouse IgG Odyssey 926-68072
Validation	DDDDK tag antibody Abcam ab49763- DDDDK tag antibody Abcam ab49763<br>ZC3HAV1 polyclonal antibody Proteintech 16820-1-AP- https://www.ptglab.com/products/ZC3HAV1-Antibody-16820-1-AP.htm<br>Anti-beta actin antibody Abcam ab6276- https://www.abcam.com/beta-actin-antibody-ac-15-ab6276.html<br>RPS6 Polyclonal Antibody Proteintech 14823-1-AP- https://www.ptglab.com/products/RPS6-Antibody-14823-1-AP.htm<br>RPL4 Monoclonal Antibody Proteintech 67028-1-Ig- https://www.ptglab.com/products/RPL4-Antibody-67028-1-Ig.htm<br>normal rabbit IgG Cell Signaling #2729- https://www.cellsignal.de/products/primary-antibodies/normal-rabbit-igg/2729#:~:text=Normal%20Rabbit%20IgG%20is%20an,Kits%20%239002%20and%20%239003.<br>RyDEN Polyclonal antibody Proteintech 27865-1-AP- https://www.ptglab.com/products/RyDEN-Antibody-27865-1-AP.htm<br>IRDye® 800CW Goat anti-Rabbit IgG Odyssey 926-32211- https://www.licor.com/bio/reagents/irdye-800cw-goat-anti-rabbit-igg-secondary-antibody<br>IRDye® 680RD Donkey anti-Mouse IgG Odyssey 926-68072<br>ZC3HAV1 polyclonal antibody Proteintech 16820-1-AP<br>Anti-beta actin antibody Abcam ab6276<br>RPS6 Polyclonal Antibody Proteintech 14823-1-AP<br>RPL4 Monoclonal Antibody Proteintech 67028-1-Ig<br>normal rabbit IgG Cell Signaling #2729<br>RyDEN Polyclonal antibody Proteintech 27865-1-AP<br>IRDye® 800CW Goat anti-Rabbit IgG Odyssey 926-32211- https://www.licor.com/bio/reagents/irdye-800cw-goat-anti-rabbit-igg-secondary-antibody<br>IRDye® 680RD Donkey anti-Mouse IgG Odyssey 926-68072- https://www.licor.com/bio/reagents/irdye-680rd-donkey-anti-mouse-igg-secondary-antibody

Eukaryotic cell lines

Policy information about cell lines

Cell line source(s)	HEK- gift from Prof. Jorg Vogel, Calu3- ATCC, Huh7- gift from Jun. Prof. Mathias Munschauer
Authentication	none of the cell lines were authenticated
Mycoplasma contamination	cells were tested by PCR and were determined to be negative for Mycoplasma
Commonly misidentified lines<br>(See ICLAC register)	*Name any commonly misidentified cell lines used in the study and provide a rationale for their use.*

Flow Cytometry

Plots

Confirm that:

- [x] The axis labels state the marker and fluorochrome used (e.g. CD4-FITC).
- [x] The axis scales are clearly visible. Include numbers along axes only for bottom left plot of group (a 'group' is an analysis of identical markers).
- [ ] All plots are contour plots with outliers or pseudocolor plots.
- [x] A numerical value for number of cells or percentage (with statistics) is provided.

Methodology

Sample preparation	HEK293 cells were transfected with the respective plasmids. 24 hours past transfection, the cells were washed with PBS and fixed with 4% PFA in PBS. After washing with PBS, the samples were used for flow cytometry measurements
Instrument	Flow cytometry was performed on a FACSAria III (BD Biosciences) or a NovoCyte Quanteon (ACEA) instrument.
Software	Flow cytometry data were analyzed with FlowJo software (BD Biosciences)
Cell population abundance	Per measurement condition at least 10,000 cells were analyzed which equates to at least 2,500 ECFP-positive cells
Gating strategy	cells were selected based on FFC and SSC. ECFP-positive (AmCyan channel) cells were analyzed for the intensity of mCherry (Texas Red channel) and EGFP (FITC channel)

- [x] Tick this box to confirm that a figure exemplifying the gating strategy is provided in the Supplementary Information.
